# Predicting academic achievement from the collaborative influences of executive function, physical fitness, and demographic factors among primary school students in China: ensemble learning methods

**DOI:** 10.1186/s12889-024-17769-7

**Published:** 2024-01-23

**Authors:** Zhiyuan Sun, Yunhao Yuan, Xuan Xiong, Shuqiao Meng, Yifan Shi, Aiguo Chen

**Affiliations:** 1 College of Physical Education, Yangzhou University, Yangzhou, 225127 China; 2 Institute of Sports, Exercise and Brain, Yangzhou University, Yangzhou, 225127 China; 3 School of Information Engineering, Yangzhou University, Yangzhou, 225127 China; 4 Department of Physical Education, Nanjing University, Nanjing, 210033 China; 5 Department of Physical Education, Xidian University, Xian, 710126 China; 6 Nanjing Sport Institute, Nanjing, 210014 China

**Keywords:** Academic achievement, Collaborative influences, Executive function, Physical fitness, Demographic information, Ensemble learning methods

## Abstract

**Background:**

Elevated levels of executive function and physical fitness play a pivotal role in shaping future quality of life. However, few studies have examined the collaborative influences of physical and mental health on academic achievement. This study aims to investigate the key factors that collaboratively influence primary school students' academic achievement from executive function, physical fitness, and demographic factors. Additionally, ensemble learning methods are employed to predict academic achievement, and their predictive performance is compared with individual learners.

**Methods:**

A cluster sampling method was utilized to select 353 primary school students from Huai'an, China, who underwent assessments for executive function, physical fitness, and academic achievement. The recursive feature elimination cross-validation method was employed to identify key factors that collaboratively influence academic achievement. Ensemble learning models, utilizing eXtreme Gradient Boosting and Random Forest algorithms, were constructed based on Bagging and Boosting methods. Individual learners were developed using Support Vector Machine, Decision Tree, Logistic Regression, and Linear Discriminant Analysis algorithms, followed by the establishment of a Stacking ensemble learning model.

**Results:**

Our findings revealed that sex, body mass index, muscle strength, cardiorespiratory function, inhibition, working memory, and shifting were key factors influencing the academic achievement of primary school students. Moreover, ensemble learning models demonstrated superior predictive performance compared to individual learners in predicting academic achievement among primary school students.

**Conclusions:**

Our results suggest that recognizing sex differences and emphasizing the simultaneous development of cognition and physical well-being can positively impact the academic development of primary school students. Ensemble learning methods warrant further attention, as they enable the establishment of an accurate academic early warning system for primary school students.

## Background

Academic success is a crucial predictor of students' future opportunities and aspirations [1]. Exceptional academic achievement not only boosts the self-confidence of primary school students [2] but also fosters a genuine interest in learning, thereby significantly contributing to their overall academic development [3]. In China, academic achievement holds immense importance as it determines the continuation of students' education, serves as an important benchmark for evaluating educational performance, and influences further educational pursuit [4]. Consequently, academic achievement has been a focal point of research within the Chinese educational landscape.

Executive function, as the core of primary school students' cognitive, emotional and social functions, has a significant impact in shaping their future quality of life [5]. Its abnormal development has been linked to various public health issues, such as autism spectrum disorders [6]. Psychological research emphasizes the critical influence of executive function on the academic achievement of primary school students [7]. Notably, primary school students with superior executive function tend to exhibit higher academic achievement [8], with the inhibition, working memory, and shifting aspects of executive function demonstrating positive correlations with academic performance [9]. Cognitive training has been shown to effectively enhance executive function and subsequently improve academic achievement [10]. Besides, physical fitness is an essential indicator of primary school students' health status, and its decline poses risks to cardiovascular health and overall future well-being [11, 12]. Existing research underscores a close association between physical fitness and academic achievement [13]. Factors such as body mass index, muscle strength, and cardiorespiratory function have proven to be reliable predictors of academic success [14–16]. Engaging in physical exercise has been identified as an effective strategy for improving academic achievement by enhancing physical fitness [17]. Additionally, demographic factors, such as sex, have been identified as contributors to variations in academic achievement [18], with girls generally outperforming boys, especially in Chinese language proficiency [19].

While executive function, physical fitness, and demographic factors have been extensively studied in relation to students' learning, it is essential to acknowledge that learning outcomes cannot be attributed solely to any individual factor. Academic achievement is a complex outcome that emerges from the interaction and integration of multiple factors operating as a cohesive unit or system [20]. In this respect, Gouveia et al. identified an interaction between physical fitness and demographic factors, indicating their joint predictive capacity for changes in the academic achievement of primary school students [21]. Another study also demonstrated that executive function acts as a fully mediating factor in the relationship between physical fitness and academic achievement [22]. However, existing research has predominantly approached the significance of executive function, physical fitness, and demographic factors from isolated perspectives, with limited exploration of their collaborative influences, emphasizing the need for further investigation in this area. In particular, it is not clearly elucidated which factors among executive function, physical fitness, and demographic variables play pivotal roles in collaboratively influencing the academic achievement of primary school students. Thus, this study will comprehensively examine multiple factors, including executive function, physical fitness, and demographic information, to identify the key factors that shape the academic achievement of primary school students.

Machine learning, a pivotal research approach in artificial intelligence, is designed to gather knowledge and patterns from intricate data, facilitating the prediction of future behaviors and trends [23]. While machine learning may pose challenges in establishing causal inferences compared to traditional statistical methods, its prowess in predicting complex data far exceeds statistical methods [24]. It focuses on achieving actual predictions and obtaining better prediction performance [25]. Over the past few years, machine learning has demonstrated remarkable success in the fields of psychology and sports science, gaining widespread recognition from researchers [26, 27]. Moreover, machine learning methods have been effectively employed in predicting academic achievement, enabling the early identification of students facing academic challenges [28]. Noteworthy applications include the use of machine learning algorithms like Naive Bayes and Decision Tree (DT) for predicting graduation grades [29], as well as the application of Support Vector Machine (SVM) and DT algorithms to distinguish students across various academic achievement groups [30]. These findings underscore the efficacy of machine learning methods in predicting academic achievement and providing timely warnings for students at risk. However, relying on individual learners based on single algorithm often struggles to capture the comprehensive relationships among variables for optimal predictive performance. To address these limitations, scholars have proposed a practical solution: ensemble learning methods [31].

Ensemble learning, a model fusion approach that combines multiple learners, commonly employs methods such as Bagging, Boosting, and Stacking [32]. Ensemble learning methods have demonstrated superior generalization ability compared to individual learners [33]. In scenarios with limited sample sizes, selecting an appropriate learner can be challenging, and ensemble methods mitigate learning risks through the collective voting of each learner [34]. Guerrero-Higueras et al. utilized interactive learning platform data to predict academic achievement, finding that Bagging and Boosting methods outperformed individual learners like Naive Bayes and Linear Discriminant Analysis (LDA) [35]. Ban et al. similarly confirmed the effectiveness of machine learning in predicting academic achievement, with the Stacking method enhancing the predictive performance of individual learners, such as DT and Logistic Regression (LR) [36]. However, further validation is necessary to ascertain whether ensemble learning methods outperform individual learners in predicting the academic achievement of primary school students using executive function, physical fitness, and demographic factors. Our research has the potential to translate findings into practical applications, offering early academic warnings for primary school students.

As previously, the academic achievement of primary school students is intricately linked to their executive function, physical fitness, and demographic factors. However, it is unclear which factors among them play key roles in collaboratively influencing the academic achievement of primary school students. Additionally, while machine learning methods have gained widespread use in predicting academic achievement, there is a need for further validation to ascertain whether ensemble learning methods outperform individual learners when predicting the academic achievement of primary school students based on executive function, physical fitness, and demographic factors. Hence, this study sought to investigate the key factors that collaboratively influence academic achievement, drawing from executive function, physical fitness, and demographic factors. Subsequently, ensemble learning methods will be employed to predict academic achievement, and their predictive performance will be compared with that of individual learners. The primary assumptions of this study are enumerated as follows:

1. Sex, body mass index, muscle strength, cardiorespiratory function, inhibitory control, working memory, and shifting factors are key factors that collaboratively influence the academic achievement of primary school students.
2. Ensemble learning methods perform better than individual learners in predicting the academic achievement of primary school students using executive function, physical fitness, and demographic factors.

The outcomes of this study will contribute additional evidence to clarify the complex relationship among executive function, physical fitness, demographic factors, and academic achievement. Moreover, this study will provide effective methods for identifying academically disadvantaged primary school students, thereby contributing valuable insights to educational practices.

## Methods

### Participants

Participants were sourced from the Physical Fitness Monitoring Project in Huai'an City, China, comprising ordinary primary school students in grades 4–6 receiving compulsory education. Utilizing the cluster sampling method, 24 test groups were selected from five schools, yielding a total of 360 primary school students. The distribution across grades included 135 students in the fourth grade, 165 students in the fifth grade, and 60 students in the sixth grade. Each group met the following criteria: (i) willingness to disclose academic achievement, (ii) participation in executive function measurement, and (iii) completion of the physical fitness test. Seven students were excluded from the analysis due to missing data, as they did not fulfill the required measurement and testing procedures. Consequently, data from 353 participants were included in subsequent processing and analysis.

### Measurements

Basic demographic factors (including sex and grade) were collected. Executive function, a multifaceted process encompassing distinct sub-functions such as inhibition, working memory, and shifting [37], was assessed using three computer-based neuropsychological assessments [38]. The stimulus presentation and response data collection were performed with the E-prime software platform. The flanker task served as an assessment of the inhibition aspect of executive function. This task comprised two trial types: congruent (e.g., LLLLL or FFFFF) and incongruent (e.g., LLFLL or FFLFF). Participants were tasked with swiftly and accurately responding to the middle letter. Evaluation indicators for the flanker task included mean reaction time and mean accuracy in both congruent and incongruent trials. To evaluate the working memory aspect of executive function, the 1-back task was employed. In this task, participants carefully observed a letter (one of B, D, L, Y, O) presented on the screen and promptly judged whether the current letter matched a previously presented one. Evaluation indicators for the 1-back task comprised mean reaction time and mean accuracy. The shifting aspect of executive function was assessed using the more-odd shifting task. This task included two trial types: homogeneous (e.g., big/small or odd/even judgment) and heterogeneous (e.g., big/small-odd/even judgment shifting). Participants were required to quickly and accurately respond to different conditions. Evaluation indicators for the more-odd shifting task included mean reaction time and mean accuracy in both homogeneous and heterogeneous trials.

We primarily consulted the Chinese National Student Physical Fitness Standard (CNSPFS) [39, 40] and selected body mass index, muscle strength, cardiopulmonary function, speed, aerobic endurance, and flexibility to evaluate the physical fitness of primary school students. Body mass index was calculated as the ratio of weight (kg) to height (m^2^). Muscle strength was assessed across three dimensions: upper limb, trunk, and lower limb. The standing long-jump test (cm) gauged lower limb strength, with participants placing their feet together behind the starting line and leaping forward as far as possible. For upper limb strength, the push-up test (times) required participants to maintain a straight body line, arms in a chest position, and hands slightly wider than shoulders, completing as many push-ups as possible. Trunk strength was evaluated through the sit-up test (times), where participants in a supine position with knees bent and feet flat completed as many sit-ups as possible within 1 min. Cardiopulmonary function was measured using the vital capacity test (ml), involving participants taking deep breaths and exhaling toward the blowing mouth until they were unable to exhale. Speed was assessed through the 50-m sprint test (s), where participants ran 50 m in a straight line on a flat playground runway at their maximum speed. Aerobic endurance was determined via the 50-m × 8 shuttle run test (s). This involved participants running back and forth between two parallel lines drawn 50 m apart, completing the course as quickly as possible four times, crossing each line with their feet. Flexibility was evaluated using the sit and reach test (cm). Participants sat on the ground, and the evaluator, ensuring their legs were straight, observed the extent of their forward reach as they slowly extended as much as possible.

### Academic achievement assessments and grouping

In China, primary school education centers around core subjects such as Chinese, mathematics, and foreign languages. Thus, we gathered the final exam scores for these three subjects and aggregated them into a total score to represent the academic achievement of primary school students. To ensure comparability, we transformed the total scores into standard scores (with an average of 0 and a variance of 1) based on school and grade parameters [41, 42]. Accordingly, primary school students whose academic achievement standard score was below the average were identified as non-high-score (NHS) students and the remaining as high-score (HS) students (56.66%, *n* = 200) [42]. In addition, to avoid possible learning risks due to different sample sizes, we randomly sampled 150 students from each group [43]. Among the 300 participants, there were 151 boys and 149 girls. The majority were in fifth grade (*n* = 136, 45.33%), followed by fourth grade (*n* = 111, 37%), and sixth grade (*n* = 53, 17.67%).

### Recursive feature elimination cross-validation

We applied the recursive feature elimination cross-validation (RFECV) method [44] to identify the key factors that collaboratively influence the academic achievement of primary school students [20]. This method involves ranking factors based on the coefficients or important properties of different models. In each iteration, it recursively eliminates a small number of redundant or less significant factors, ultimately retaining the optimal set of factors. Additionally, to mitigate potential collinearity impacts on predictive performance, we initially excluded highly correlated factors. Correlation coefficients between each pair of factors were computed using Pearson and Spearman correlation analyses. In instances where the absolute value of the correlation coefficient exceeded 0.7, only one factor was retained.

### Single machine learning algorithms

A published systematic review highlighted the significant contributions of SVM and DT algorithms as individual models in predicting academic achievement using machine learning methods, and the LR algorithm was also reported to have good predictive performance [45]. In addition, the LDA algorithm has been proven to be applicable for academic achievement prediction [35]. Hence, we opted for four single algorithms—SVM, DT, LR, and LDA—to establish individual learners for the prediction of academic achievement.

The SVM algorithm is employed for classifying academic achievement by identifying the optimal hyperplane with the largest classification margin. Notably, it incorporates a diverse range of powerful kernel functions, enabling the processing of datasets using highly complex structured methods [46]. On the other hand, the DT algorithm excels at summarizing decision rules from datasets with features and labels, presenting these rules in the form of a tree graph. Recognized for its ease of understanding, suitability for various types of data, and wide applications across fields, the DT algorithm is a versatile tool [47]. LR algorithms are designed to predict the probability of future outcomes based on existing data performance. With a regularization term integrated into the model, LR can effectively reduce model complexity and counteract overfitting concerns [48]. Lastly, the LDA algorithm plays a pivotal role in projecting data into a low-dimensional space. It aims to maximize the differences between categories while minimizing differences within categories, thus effectively achieving the objectives of classification and discrimination [49].

### Ensemble learning methods

Ensemble learning methods, including Bagging, Boosting, and Stacking, have made remarkable contributions to predicting academic achievement [45]. The Bagging method [50] enhances predictive performance by reducing classification error variance. A notable member of the Bagging category is the Random Forest (RF) algorithm [51], which has demonstrated robust predictive capabilities in academic achievement [52]. Bagging employs the Bootstrap method to randomly select M samples from the primary school students' dataset, with replacement, ensuring eligibility for subsequent sampling. This process iterates to train N weak classifiers, and their outputs are combined, each receiving equal weighting.

Boosting is another ensemble method that elevates weak classifiers to robust classifiers. The eXtreme Gradient Boosting (XGB) is a powerful member of the Boosting family [53] and has been applied successfully in predicting academic achievement [54]. The boosting process starts by training a weak classifier using the dataset. It then adjusts the sample distribution based on the weak classifier's performance, assigning greater weight to previously misclassified samples. The next weak classifier is then trained according to the adjusted sample distribution [55]. Finally, these weak classifiers are combined, with those performing better assigned higher weights.

**Fig. 1:**
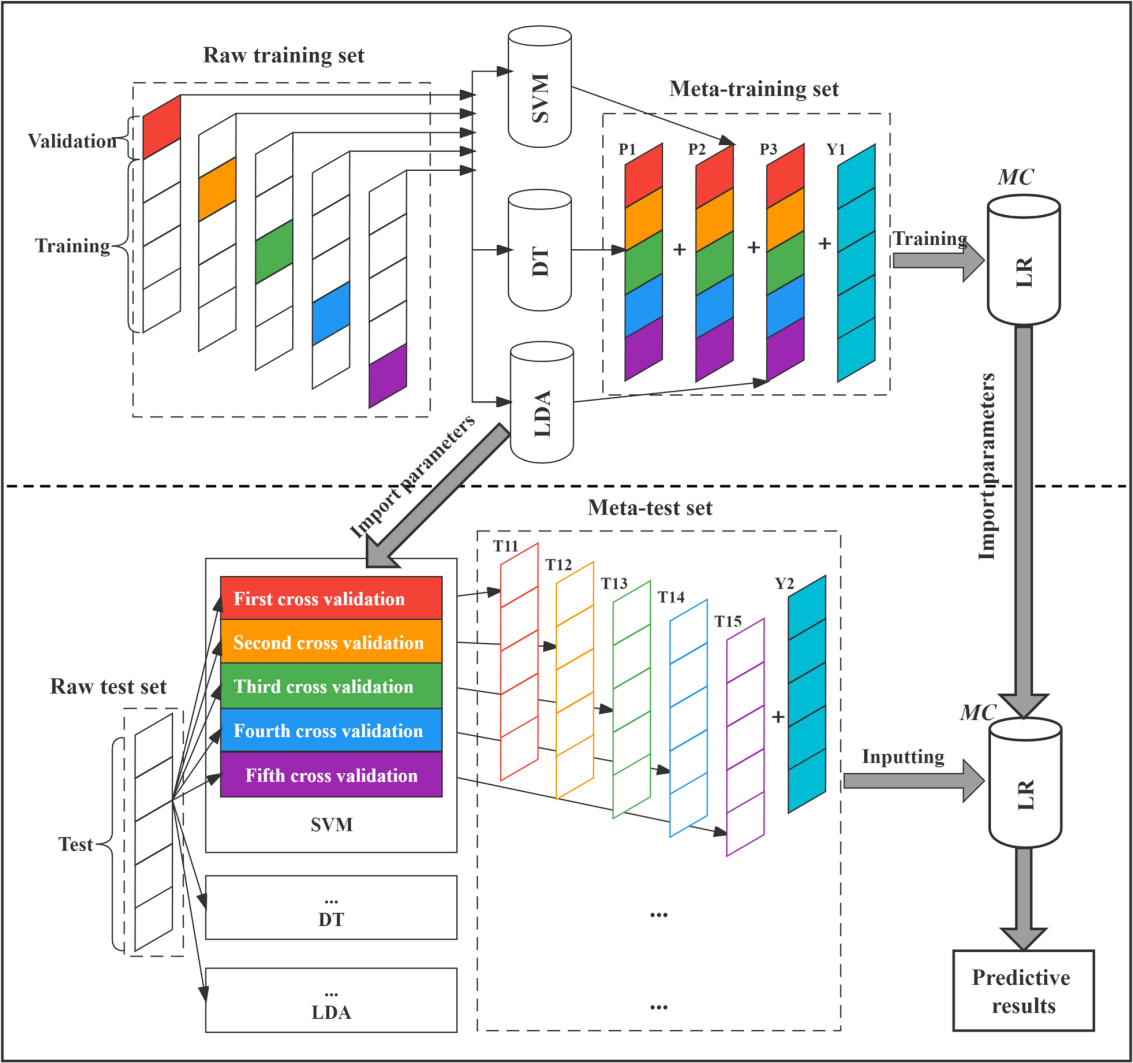
The principle of Stacking ensemble learning model Note. SVM is Support Vector Machine; DT is Decision Tree; LDA is Linear Discriminant Analysis; LR is Logistic Regression; MC is Meta-Classifier

The Stacking method [56] constructs several base classifiers using the dataset. It generates a meta-dataset and passes it to the next layer, where the meta-classifier of the final layer produces the ultimate prediction outcome. The outputs of the base classifiers within the meta-dataset are considered new features, while the raw dataset labels are retained as sample labels. For the stacking ensemble learning method in this study, four selected single algorithms—SVM, DT, LR, and LDA—were used to establish a Stacked ensemble learning model for predicting academic achievement (Fig. 1).

#### Evaluation and validation

The dataset was randomly divided into a training set (80%, *n* = 240) and a test set (20%, *n* = 60) to facilitate machine learning model training and validation. To enhance predictive performance reliability, we employed the repeated five-fold cross-validation method. This method involves randomly dividing the training set into five folds, with four folds used for training in each iteration and the remaining fold reserved for validation. The average accuracy following cross-validation was reported as an assessment of predictive performance [57]. Accuracy is defined as the proportion of correctly classified samples and is specifically expressed as:

$$Accuracy=({\text{TP}}+{\text{TN}})/({\text{TP}}+{\text{FP}}+{\text{FN}}+{\text{TN}})$$

where TP (true positive) refers to the number of samples whose actual value is positive, and the model predicts them as positive. TN (true negative) is the number of samples whose actual value is negative, and the model predicts them as negative. FP (false positive) is the number of samples whose actual value is negative, and the model predicts them as positive. FN (false negative) is the number of samples whose actual value is positive, and the model predicts them as negative.

Besides, the trained models were applied to the test samples, and multiple indicators such as accuracy, precision, and recall were used to re-evaluate predictive performance [45]. Precision and recall are defined as follows:

$$Precision={\text{TP}}/({\text{TP}}+{\text{FP}})$$

$$Recall={\text{TP}}/({\text{TP}}+{\text{FN}})$$

The accuracy, precision, and recall indicators ranged from 0 to 1, with values closer to 1 indicating better predictive performance. Additionally, the permutation test method was applied to measure the probability that the final predicted results occurred by chance.

## Results

### Key factors

**Fig. 2:**
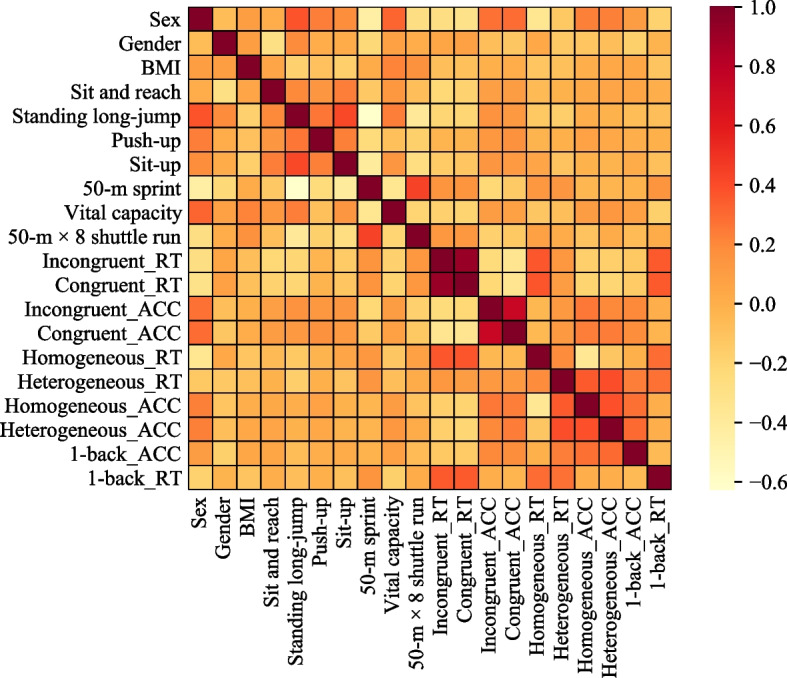
The results of correlation analysis for factors Note. Incongruent_RT and Incongruent_ACC are the mean reaction time and accuracy in incongruent trials, respectively. Congruent_RT and Congruent_ACC are the mean reaction time and accuracy in congruent trials. Homogeneous_RT and Homogeneous_ACC are the mean reaction time and accuracy in homogeneous trials. Heterogeneous_RT and Heterogeneous_ACC are the mean reaction time and accuracy in heterogeneous trials. 1-back_RT and 1-back_ACC are the mean reaction time and accuracy in 1-back task

To mitigate potential collinearity effects, we excluded highly correlated factors based on the results of Pearson and Spearman correlation analyses. As illustrated in Fig. 2, the average reaction time and accuracy in congruent trials exhibited strong correlations with those in incongruent trials, with correlation coefficients exceeding 0.7. Consequently, we excluded the mean reaction time and accuracy in incongruent trials from further analysis. In total, 18 factors were retained for subsequent analysis, encompassing sex, grade, body mass index, sit and reach, standing long-jump, push-up, sit-up, 50-m sprint, vital capacity, 50-m × 8 shuttle run, and the mean reaction time and accuracy in congruent trials, homogeneous trials, heterogeneous trials, and 1-back task.

**Fig. 3:**
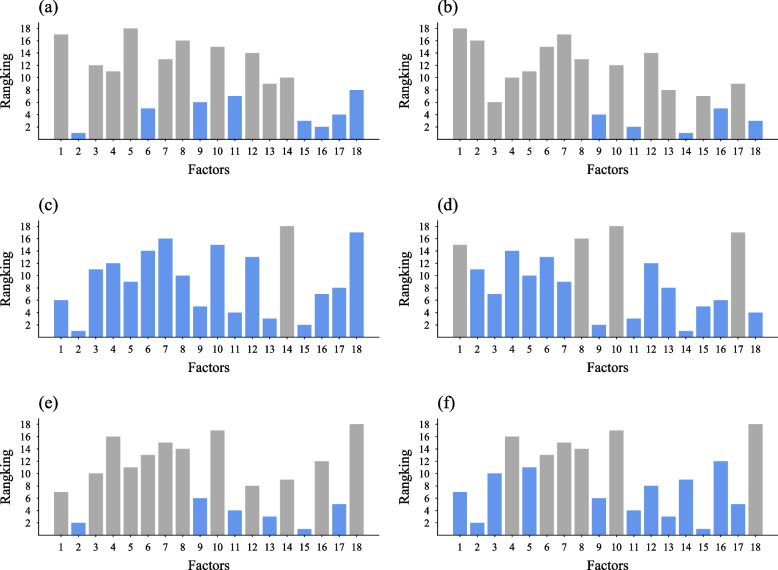
The best factor subset and feature importance ranking of each algorithm Note. **a** represents the eXtreme Gradient Boosting algorithm; **b** is the Random Forest algorithm; **c** is the Support Vector Machine algorithm; **d** is the Decision Tree algorithm; **e** is the Logistic Regression algorithm; **f** is the Linear Discriminant Analysis algorithm. Blue color indicates the factors selected as key, while gray indicates exclusion. Due to space constraints, all factor names couldn't be labeled in the diagram. The factors in each subfigure are in the same order: sex, grade, body mass index, sit and reach, standing long-jump, push-up, sit-up, 50-m sprint, vital capacity, 50-m × 8 shuttle run, mean reaction time in congruent trials, mean accuracy in congruent trials, mean reaction time in homogeneous trials, mean reaction time in heterogeneous trials, mean accuracy in homogeneous trials, mean accuracy in heterogeneous trials, mean reaction time in 1-back task, and mean accuracy in 1-back task

We utilized the RFECV method to investigate the key factors that collaboratively influence primary school students' academic achievement. In Fig. 3(a), the XGB algorithm identified a crucial factor set consisting of eight factors. Sex, push-up, vital capacity, mean reaction time in congruent and 1-back task, and mean accuracy in homogeneous and heterogeneous trials were identified as influential factors. Notably, sex was ranked as the most significant factor.

In Fig. 3(b), the RF algorithm selected a significant factor set comprising five factors. Vital capacity, mean reaction time in congruent trials, heterogeneous trials, and 1-back task, along with mean accuracy in heterogeneous trials, were identified as substantial contributors to academic achievement. Mean reaction time in heterogeneous trials was considered the most crucial.

Figure 3(c) illustrates that the SVM algorithm chose a factor set of 17 factors, excluding only the mean reaction time in heterogeneous trials. Sex was identified as the most critical factor.

Figure 3(d) displays that the DT algorithm opted for a factor set of 14 factors. Grade, 50-m sprint, 50-m × 8 shuttle run, and mean accuracy in 1-back task were not considered key factors. Mean reaction time in heterogeneous trials was regarded as the most important.

In Fig. 3(e), the LR algorithm determined that the optimal factor set should include six factors. Sex, vital capacity, mean reaction time in congruent and homogeneous trials, and mean accuracy in homogeneous trials and 1-back task played pivotal roles. Mean accuracy in homogeneous trials was identified as the most important.

As shown in Fig. 3 (f), the LDA algorithm determined the optimal factor set, which included 12 factors. This algorithm excluded sit and reach, push-up, sit-up, 50-m sprint, 50-m × 8 shuttle run, and mean reaction time in 1-back task. It considered mean accuracy in homogeneous trials as the most important factor.

The results indicated that sex, body mass index, muscle strength, cardiorespiratory function, inhibition, working memory, and shifting were considered key factors by no less than half of the machine learning algorithms. Additionally, the shifting aspect of executive function was identified as the most important factor by most algorithms.

### Predictive performance

We constructed two ensemble learning models, XGB and RF, using the training set. Additionally, SVM, DT, LR, and LDA models were established, and a Stacking ensemble learning model was created based on their prediction results. We utilized the cross-validation method to obtain ten predictive accuracy for each model and compared their average accuracy. Table 1 displays the accuracy of seven machine learning models in predicting primary school students' academic achievement using their optimal feature subset.

**Table 1:** The accuracy of machine learning models in the training set (%)

Subsets	XGB	RF	SVM	DT	LR	LDA	Stacking
*5-fold CV (1st)*							
First	72.92	75	66.67	62.5	66.67	62.5	64.58
Second	64.58	62.5	66.67	56.25	68.75	66.67	75
Third	62.5	60.42	60.42	64.58	62.5	60.42	68.75
Fourth	72.92	72.92	54.17	58.33	68.75	54.17	62.5
Fifth	79.17	79.17	62.5	64.58	52.08	72.92	70.83
*5-fold CV (2nd)*							
First	68.75	62.5	58.33	60.42	54.17	64.58	66.67
Second	75	75	58.33	68.75	60.42	64.58	68.75
Third	70.83	79.17	62.5	58.33	58.33	62.5	72.92
Fourth	70.83	66.67	58.33	64.58	60.42	68.75	64.58
Fifth	64.58	70.83	70.83	56.25	62.5	56.25	68.75
*Repeated fivefold CV*							
Average	70.21	70.42	61.88	61.46	61.46	63.33	68.33

CV is cross-validation. XGB is eXtreme Gradient Boosting model; RF is Random Forest model; SVM is Support Vector Machine model; DT is Decision Tree model; LR is Logistic Regression model; LDA is Linear Discriminant Analysis model; Stacking is Stacking ensemble learning model

As shown in Table 1, the average accuracy of XGB, RF, SVM, DT, LR, LDA, and Stacking models was 70.21% (permutation test, iterations = 1000, *p* < 0.001), 70.42% (*p* < 0.001), 61.88% (*p* < 0.001), 61.46% (*p* < 0.001), 61.46% (*p* < 0.001), 63.33% (*p* < 0.001), and 68.33% (*p* < 0.001), respectively. The highest accuracy, occurring three times, was 79.17%, with two instances in the RF model and one in the XGB model. The lowest accuracy, recorded once, was 52.08% in the LR model. The minimum accuracy of XGB, RF, SVM, DT, LR, LDA, and Stacking models exceeded the baseline accuracy (50%), with the highest accuracy exceeding it by 29.17%, 29.17%, 20.83%, 18.75%, 18.75%, 22.92%, and 25%, respectively. Notably, the lowest, highest, and average accuracy of the three ensemble learning models exceeded those of the four individual learners, suggesting that ensemble learning methods outperform traditional machine learning methods. The results indicated that machine learning models could predict academic achievement of primary school students using executive function, physical fitness, and demographic factors, with the accuracy of ensemble learning models surpassing that of individual learners.

**Fig. 4:**
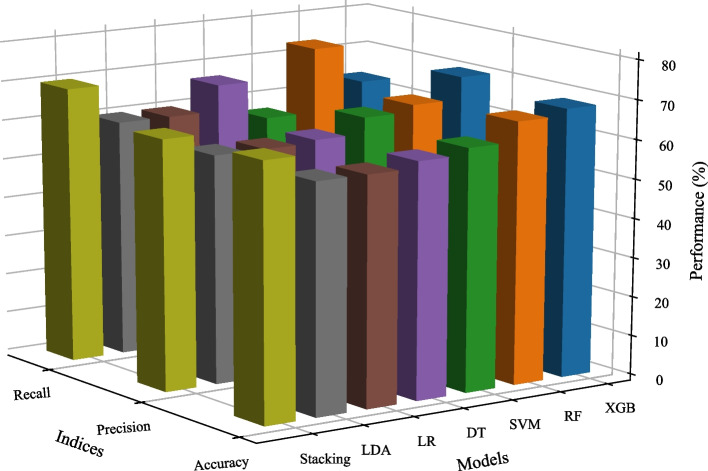
The performance of machine learning models in test samples Note. XGB is eXtreme Gradient Boosting model; RF is Random Forest model; SVM is Support Vector Machine model; DT is Decision Tree model; LR is Logistic Regression model; LDA is Linear Discriminant Analysis model; Stacking is Stacking ensemble learning model

We inputted test samples into the machine learning models and used accuracy, precision, and recall indicators to re-evaluate their prediction performance, directly confirming their generalization capability. In Fig. 4, the XGB model achieved accuracy, precision, and recall of 68.33%, 70.37%, and 63.33% (*ps* = 0.004), respectively. The RF model achieved 66.67%, 64.71%, and 73.33% (*ps* = 0.003), while the Stacking model achieved 65%, 63.64%, and 70% (*ps* = 0.023). In addition, the evaluation indices scores of the three ensemble learning models in the test samples were generally higher than those of the four individual learners. The results once again validated that the ensemble learning model yielded stronger performance than individual learners in predicting the academic achievement of primary school students.

## Discussion

We sought to determine which factors from executive function, physical fitness, and demographic factors play key roles in collaboratively influencing the academic achievement of primary school students. Our findings indicated that sex, body mass index, muscle strength, cardiorespiratory function, inhibition, working memory, and shifting were key factors that collaboratively influence the academic achievement of primary school students. To begin with, we identified sex as a key demographic factor associated with academic achievement. Previous studies have demonstrated sex-based differences in academic performance [18]. This discrepancy may be attributed to girls having higher failure anxiety than boys, potentially leading to reduced academic success [58].

Besides, physical fitness-related factors, including body mass index, muscle strength, and cardiorespiratory function, emerged as significant contributors to academic achievement in primary school students. Firstly, Castelli et al. observed an inverse relationship between the overall academic achievement of third and fifth-grade students and their body mass index [59]. Research findings imply that elevated body mass index may contribute to issues of overweight/obesity, heightening the risk of unequal treatment and limiting access to learning resources for primary school students [60]. Secondly, Xu et al. found positive correlations between the upper limb strength, lower limb strength, and trunk strength of fifth-grade students with their academic achievement [42]. Scientific evidence supports the notion that strength training can enhance brain plasticity and various functions, including learning and memory [61]. Thirdly, Liang et al. uncovered a connection between poor vital capacity and subpar academic achievement [16]. A growing body of research suggests that aerobic exercise improves cardiorespiratory fitness, inducing changes in brain structure and function [62]. These alterations positively affect executive function [63], which is necessary for academic improvement.

Finally, executive function-related factors, namely inhibition, working memory, and shifting, were recognized as pivotal elements influencing academic achievement. Borella et al. employed Stroop task to assess the inhibition aspects of executive function and determined that inhibition could serve as a predictor for the academic achievement of primary school students [64]. A meta-analysis uncovered that primary school students with lower academic achievement exhibited poorer performance in working memory task compared to their higher-achieving peers [65]. Magalhães et al. further demonstrated that the shifting aspects of executive function could be predictive of academic achievement [66]. Another study found that persistent errors in the Wisconsin Card Sorting test inversely predicted mathematical ability [67]. The predictive influence of executive function on the academic achievement of primary school students should not come as a surprise. As an advanced cognitive process, executive function represents a critical cognitive ability essential for robust academic performance. Moreover, executive control is intricately linked to the functioning of the prefrontal cortex [68], a region closely associated with learning and memory processes.

Additionally, we conducted an exploration to assess whether ensemble learning methods could outperform individual learners in predicting the academic achievement of primary school students, considering executive function, physical fitness, and demographic factors. Our findings revealed that the accuracy of the XGB, RF, and Stacking models surpassed that of individual learners, a result consistently verified through test samples. Ding et al. used factors such as mental health status and coping styles to predict academic achievement [69]. Their results demonstrated that machine learning methods could accurately predict academic success, with Bagging exhibiting superior predictive performance compared to Naive Bayes and K-Nearest Neighbors algorithms. Another investigation collected demographic and personality factors to predict academic achievement [70]. The outcomes suggested that machine learning models could accurately forecast students' academic success, with the Stacking model demonstrating superior performance over individual learners. Ensemble learning operates on the principle of combining multiple weak learners to create a strong learner. Bagging employs the Bootstrap method to generate each base learner from a random subset of the dataset. Boosting establishes multiple base learners by adjusting sample weights and assigning higher weights to those with superior performance. Stacking selects heterogeneous individual learners as base learners, allowing them to observe data from various data spaces and structures. Ensemble learning methods employ diverse strategies to mitigate the predictive errors of base learners, ultimately enhancing predictive performance and generalization capabilities.

Our findings yield profound insights for the field of education, poised to instigate positive changes in practice. In practical applications, this study establishes a cornerstone for personalized education. Schools can forge customized learning plans by consistently assessing key factors, thereby better catering to the individual needs of each primary school student. Additionally, this research strengthens the potential for early intervention measures. Educators can promptly identify academic challenges faced by primary school students and offer targeted support, fostering an environment where students can more effectively realize their potential [28]. In terms of education policy, our study prompts contemplation on resource allocation and policy formulation. Governments and school administrators, armed with an understanding of key factors, can allocate resources more precisely to ensure schools provide effective support and education. Furthermore, these findings may provoke considerations among policymakers, leading to adjustments that better integrate data science into educational practices and propel the education system toward a more intelligent and personalized direction [71]. In summary, this research makes substantial contributions to the field of education, laying a robust foundation for advancing the academic development and personalized learning of primary school students. It offers education policymakers a fresh perspective, inspiring potential adjustments in policies to better align with the needs of primary school students and optimize the entire education system.

It is crucial to acknowledge the limitations inherent in our study. Firstly, while past research has effectively utilized smaller datasets to construct machine learning models for accurate academic achievement prediction [72], and ensemble learning methods are deemed suitable for situations with smaller samples [34], it remains imperative to conduct further research on more extensive datasets. Moreover, our study exclusively focused on executive function, physical fitness, and demographic factors as predictive variables. To enhance the predictive performance of our models, it is worthwhile to explore additional avenues. One plausible approach involves the inclusion of variables from diverse categories, such as cortical thickness [73] and hippocampal volume [74]. This strategy aims to encompass a broader spectrum of factors associated with academic achievement, leveraging the robust capabilities of machine learning methods for intricate data analysis and, consequently, improving predictive performance.

## Conclusion

Our findings indicated that sex, body mass index, muscle strength, cardiorespiratory function, inhibition, working memory, and shifting were key factors that collaboratively influence the academic achievement of primary school students. Additionally, ensemble learning models demonstrated superior performance compared to individual learners in predicting academic achievement of primary school students. These findings underscore the importance of recognizing sex differences and highlighting the interconnected development of cognition and the body, which can positively impact the academic development of primary school students. Moreover, our results advocate for greater attention to ensemble learning methods, emphasizing their utility in establishing an accurate academic early warning system for primary school students.

## Data Availability

The datasets used and/or analysed during the current study are available from the corresponding author on reasonable request.

## Abbreviations

NHS: Non-High-Score
HS: High-Score
DT: Decision Tree
SVM: Support Vector Machine
LDA: Linear Discriminant Analysis
LR: Logistic Regression
RF: Random Forest
XGB: eXtreme Gradient Boosting
RFECV: Recursive Feature Elimination Cross-Validation
